# In emergently ventilated trauma patients, low end-tidal CO_2_ and low cardiac output are associated and correlate with hemodynamic instability, hemorrhage, abnormal pupils, and death

**DOI:** 10.1186/1471-2253-13-20

**Published:** 2013-09-11

**Authors:** C Michael Dunham, Thomas J Chirichella, Brian S Gruber, Jonathan P Ferrari, Joseph A Martin, Brenda A Luchs, Barbara M Hileman, Renee Merrell

**Affiliations:** 1 Trauma/Critical Services, Level I Trauma Center, St. Elizabeth Health Center, 1044 Belmont Avenue, Youngstown, OH 44501, USA

**Keywords:** Cardiac output, Trauma, Validation, Capnography

## Abstract

**Background:**

In a smaller experience, the authors previously demonstrated that end-tidal carbon dioxide (PetCO_2_) and cardiac output (CO) had a positive association in emergently intubated trauma patients during Emergency Department resuscitation. The aim of this larger study was to reassess the relationship of PetCO_2_ with CO and identify patient risk-conditions influencing PetCO_2_ and CO values.

**Methods:**

The investigation consists of acutely injured trauma patients requiring emergency tracheal intubation. The study focuses on the prospective collection of PetCO_2_ and noninvasive CO monitor (NICOM®) values in the Emergency Department.

**Results:**

From the end of March through August 2011, 73 patients had 318 pairs of PetCO_2_ (mm Hg) and CO (L/min.) values. Mean data included Injury Severity Score (ISS) ≥15 in 65.2%, Glasgow Coma Score of 6.4 ± 4.6, hypotension in 19.0%, and death in 34.3%. With PetCO_2_ ≤ 25 (15.9 ± 8.0), systolic blood pressure was 77.0 ± 69, CO was 3.2 ± 3.0, cardiac arrest was 60.4%, and mortality was 84.9%. During hypotension, CO was lower with major blood loss (1.9), than without major loss (5.0; *P* = 0.0008). Low PetCO_2_ was associated with low CO (*P* < 0.0001). Low PetCO_2_ was associated (*P* ≤ 0.0012) with ISS > 20, hypotension, bradycardia, major blood loss, abnormal pupils, cardiac arrest, and death. Low CO was associated (*P* ≤ 0.0059) with ISS > 20, hypotension, bradycardia, major blood loss, abnormal pupils, cardiac arrest, and death.

**Conclusions:**

During emergency department resuscitation, a decline in PetCO_2_ correlates with decreases in noninvasive CO in emergently intubated trauma patients. Decreasing PetCO_2_ and declining NICOM CO are associated with hemodynamic instability, hemorrhage, abnormal pupils, and death. The study indicates that NICOM CO values are clinically discriminate and have physiologic validity.

## Background

The authors have previously established clinical validity of the noninvasive cardiac output monitor (NICOM®), in trauma activation patients [1]. In that investigation, the authors demonstrated that end-tidal carbon dioxide (PetCO_2_) and noninvasive cardiac output (CO) values had a significantly positive relationship. The current study represents a larger cohort of consecutive, emergently intubated trauma patients.

An extensive search of literature revealed that no other trauma investigation describes the relationship of post-injury CO and PetCO_2_. However, research involving non-trauma patients [2-5] and animals [6-8] indicates that CO and PetCO_2_ decrease during hemodynamic instability and are statistically correlated.

We hypothesized that CO and PetCO_2_, in this larger group of patients, would also have a positive and significant association. We further conjectured that clinical risk-conditions that had a significant relationship with CO would also influence PetCO_2_ values.

## Methods

This study was approved by the St. Elizabeth Health Center Institutional Review Board. The Review Board waived the need for informed patient consent. This is a study of emergently intubated trauma patients at a Level I trauma center with prospective documentation of PetCO_2_ and NICOM® in the Emergency Department. The investigation included patients managed by the trauma service during March 22 through August 17, 2011. NICOM® monitoring (Cheetah Medical Inc, Vancouver, WA), using Bioreactance® technology, was initiated as soon as possible after Emergency Department arrival. NICOM® sensors were applied to the torso by surgical residents and nurses entered patient information into the NICOM® device and initiated monitor calibration. The respiratory therapist inserted a continuous capnography sensor between the endotracheal tube and ventilator tubing.

The nursing staff documented the following parameters on the emergency department trauma flow sheet: heart rate, systolic blood pressure (BP), diastolic BP, CO, and PetCO_2_. PetCO_2_ and CO were documented approximately every 10 minutes on the flow sheet with the simultaneous heart rate, systolic BP, and diastolic BP. For patients who presented with asystole, the heart rate, and blood pressure were documented as zero values by the nurse. In this circumstance, the NICOM device was not in place, however, we assigned a CO value of 0 L/min. In cases of cardiac arrest, cardiopulmonary resuscitation was initiated and included chest compressions. Within 48 hours, flow sheets were reviewed and all paired CO and PetCO_2_ values, along with systolic BP, diastolic BP, and heart rate, were entered into an electronic database.

A Blood Loss Form was devised to categorize estimates of blood loss (none, < 500 mL, 500-1000 mL, or > 1000 mL), based on diagnostic imaging, estimated external blood loss, and/or operative findings. Using the above scheme, estimates of blood loss were documented for each of the six following sources: external, hemothorax, hemoperitoneum, pelvic ring disruption hematoma, retroperitoneal hematoma, and subcutaneous hemorrhage. The presence or absence of femoral fracture with major thigh swelling was documented for each patient. The presence of abnormal pupils was also documented by the surgical resident on the blood loss form. The senior-level surgical resident completed the Blood Loss Form within six hours of Emergency Department arrival. Major blood loss was defined as a femoral fracture with major thigh swelling or ≥ 500 mL loss from an external source, hemoperitoneum, hemothorax, pelvic hemorrhage, retroperitoneal hematoma, or subcutaneous hemorrhage.

Hypotension was defined as systolic BP < 100 and bradycardia was defined as a heart rate < 60. Red blood cell (RBC) transfusion was the units transfused within the first three hours of Emergency Department arrival. The Glasgow Coma Score was documented on Emergency Department arrival. The Injury Severity Score (ISS) was obtained from the Trauma Registry. Age, base deficit, lactate, Glasgow Coma Score, and RBC transfusion amounts came from the medical record.

We evaluated the statistical relationship between CO and PetCO_2_. We separately evaluated the relationships of CO with the following clinical conditions: high ISS, hypotension, bradycardia, major blood loss, any RBC transfusion, abnormal pupils, cardiac arrest, and death. We repeated the relationship analysis with PetCO_2_ using the same clinical conditions. CO was dichotomized into two categories, < 4.5 L/min and ≥ 4.5 L/min. This threshold CO value was selected because it represented two categories where the difference between the mean PetCO_2_ values for the two groups was maximal (lowest *P*-value). ISS was also dichotomized into two categories, ≤ 20 and > 20. This threshold value was selected because it represented two categories where the PetCO_2_ and CO mean differences between the two groups were maximal. SAS System for Windows, release 9.2 (SAS Institute Inc., Cary, NC, USA), was used to perform the statistical analysis. *P* < 0.05 represented statistical significance.

## Results

During the study period, 73 emergently intubated patients had 318 PetCO_2_ (mm Hg) and CO (L/min) paired-values in the emergency department. All patients had PetCO_2_ monitoring in the emergency department (total values documented were 407). CO was available for 64 (87.7%) patients, using NICOM monitor values and/or an assumed CO of 0 L/min when asystole was present on admission. Traits of the study patients are in Table 1 and indicate that severe anatomic injuries, hemodynamic instability, hemorrhage, metabolic acidosis, abnormal pupils, and death were common.

**Table 1:** Emergently intubated patient traits (n = 318 observations)

	
**PetCO**_**2 **_**(mmHg)**	**29.9 ± 10.0**
**Cardiac Output (L/min)**	6.0 ± 2.6
**Age**	46.7 ± 18.3
**ISS**	21.5 ± 13.9
**ISS ≥ 15**	65.2%
**Hypotension**	18.5%
**Bradycardia**	13.4%
**Cardiac Arrest**	9.1%
**Major Blood Loss**	23.3%
**RBC Transfusion**	23.3%
**Lactate (mmol/L)**	4.4± 3.5
**Base Deficit**	−4.8 ± 7.4
**GCS**	6.4 ± 4.6
**GCS ≤ 8**	72.6%
**Abnormal Pupils**	34.3%
**Death**	34.3%

*PetCO*_*2*_ end-tidal carbon dioxide, *ISS* Injury Severity Score, *GCS* Glasgow Coma Score.

Of the patients with hypotension, 39.2% had no major blood loss. In hypotensive patients, CO was lower with major blood loss (1.9 ± 3.0) when compared to those without major blood loss (5.0 ± 2.9; *P* = 0.0008). In hypotensive patients, PetCO_2_ was lower with major blood loss (15.2 ± 11.7) when compared to those without major blood loss (24.8 ± 10.1; *P* = 0.0005). With bradycardia, the heart rate was 13.8 ± 25.4 (0-59). Of the patients with abnormal pupils, cardiac arrest occurred in 14.8% and no cardiac arrest in 85.2%.

**Figure 1:**
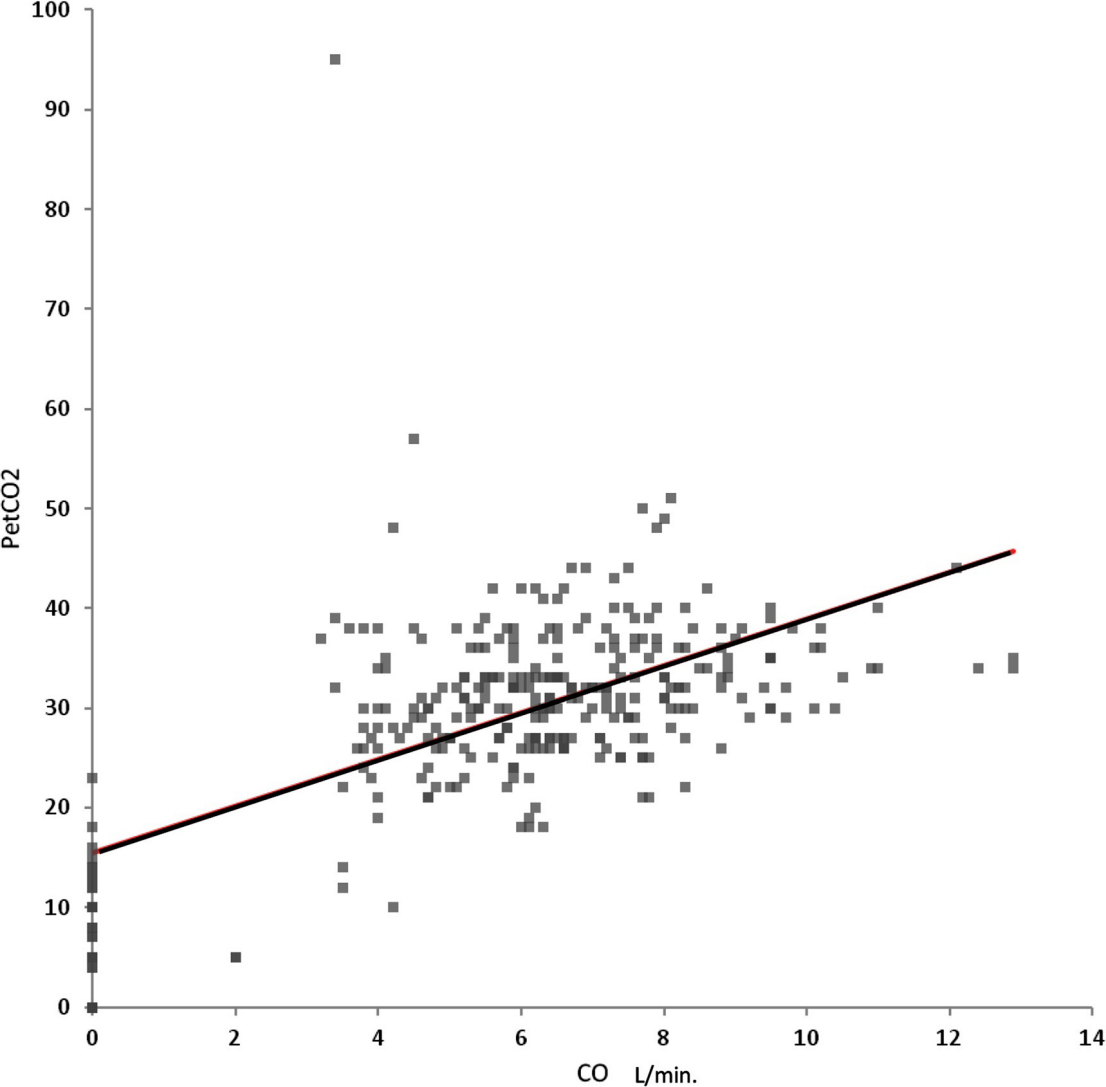
**Scatter plot of cardiac output and PetCO**_**2**_**.**

PetCO_2_ was significantly lower with ISS > 20, hypotension, bradycardia, cardiac arrest, major blood loss, RBC transfusion, abnormal pupils, and death (Table 2). PetCO_2_ had a significant inverse correlation with ISS (*P* = 0.0006). The ISS > 20 patients had more hypotension (P = 0.018), greater blood loss (P < 0.0001), and a greater need for RBC transfusion (P < 0.0001). When compared to patients with normal pupils, those with abnormal pupils had lower CO (*P* = 0.0059), more hypotension (*P* = 0.0103), and lower admission GCS (4.2 ± 2.2) (*P* < 0.0001). With asystole, PetCO_2_ was zero in 6 of 29 observations. Low CO was also associated with ISS > 20, hypotension, bradycardia, cardiac arrest, major blood loss, RBC transfusion, abnormal pupils, and death (Table 3). CO had a significant inverse correlation with ISS (*P* = 0.0002). PetCO_2_ was significantly decreased with CO < 4.5 L/min (Table 2). Low PetCO_2_ was associated with low CO (*r* = 0.60; *P* < 0.0001). A scatter plot of this relationship is in Figure 1. After excluding asystole patients with presumed CO = 0 L/min, the association between PetCO_2_ and CO remained highly significant (*P* < 0.0001).

**Table 2:** **Decreasing PetCO**_**2 **_**with clinical risk-conditions**

	**PetCO**_**2 **_**(mmHg)**		
**Condition:**	**Present**	**Absent**	***P*-value**
**Cardiac Output < 4.5 L/min**	19.5 ± 8.7	31.9 ± 5.9	< 0.0001
**Injury Severity Score > 20**	28.7 ± 9.4	32.5 ± 9.3	0.0012
**Hypotension**	18.9 ± 12.0	32.5 ± 7.4	< 0.0001
**Bradycardia**	17.6 ± 12.2	31.9 ± 8.1	< 0.0001
**Cardiac Arrest**	8.4 ± 7.9	31.6 ± 8.1	< 0.0001
**Major Blood Loss**	23.1 ± 12.8	31.7 ± 8.3	< 0.0001
**RBC Transfusion**	22.8 ± 10.6	32.1 ± 8.7	< 0.0001
**Abnormal Pupils**	27.4 ± 11.1	31.4 ± 9.0	0.0002
**Death**	24.4 ± 13.5	31.6 ± 7.5	< 0.0001

*PetCO*_*2*_ end-tidal carbon dioxide, *RBC* Red Blood Cell.

**Table 3:** Decreasing cardiac output with clinical risk-conditions

	**Cardiac output (L/min)**		
**Condition:**	**Present**	**Absent**	***P*-value**
**Injury Severity Score > 20**	5.6 ± 2.3	7.0 ± 2.1	< 0.0001
**Hypotension**	2.8 ± 3.3	6.7 ± 1.8	< 0.0001
**Bradycardia**	2.2 ± 2.9	6.7 ± 1.8	< 0.0001
**Cardiac Arrest**	0.0 ± 0.0	6.6 ± 1.8	< 0.0001
**Major Blood Loss**	4.2 ± 3.7	6.5 ± 1.9	< 0.0001
**RBC Transfusion**	4.5 ± 3.5	6.4 ± 2.2	0.0001
**Abnormal Pupils**	5.4 ± 2.7	6.3 ± 2.5	0.0059
**Death**	3.9 ± 3.1	6.7 ± 1.9	< 0.0001

*RBC* Red Blood Cell.

Independent, simultaneous variable associations with PetCO_2_ (*P* ≤ 0.004; r^2^ = 0.48) included bradycardia, RBC transfusion, abnormal pupils, and CO. With PetCO_2_ ≤ 25 mmHg (15.9 ± 8.0), systolic BP mmHg was 77.0 ± 69.0, CO was 3.2 ± 3.0 L/min, cardiac arrest rate was 60.4%, and mortality rate was 84.9%. Using all 407 PetCO_2_ values, greater relevant details are displayed in Table 4, indicating that patients with lower PetCO_2_ had higher cardiac arrest rates and lower CO, systolic BP, and heart rate. PetCO_2_ was higher for the surviving patients (31.6 ± 7.5); when compared to the dying patients (24.4 ± 13.5; *P* < 0.0001). Mortality was 67.1% with PetCO_2_ ≤ 25; 13.1% with PetCO_2_ 26-35, and 34.0% with PetCO_2_ ≥ 36. The mortality rates were significantly different (*P* < 0.0001) for each of the three inter-group comparisons. Of the patients with PetCO_2_ ≥ 36, none were in cardiac arrest in the Emergency Department and the majority of deaths occurred on a date occurring after the day of admission.

**Table 4:** **Select physiologic variables and mortality outcomes according to PetCO**_**2**_

**PetCO**_**2**_	**No.**	**Arrest**	**CO**	**Sys. BP**	**Heart rate**	**Mortality**
**0–15**	38	71.1%	0.5 ± 1.2	20 ± 36	30 ± 52	100.0%
**16–20**	10	20.0%	4.3 ± 2.8	86 ± 55	53 ± 32	40.0%
**21–25**	37	2.7%	5.6 ± 1.8	131 ± 51	83 ± 24	40.5%
**26–30**	111	0.0%	6.2 ± 1.6	137 ± 33	95 ± 25	15.3%
**31–35**	111	0.0%	7.1 ± 1.9	144 ± 30	93 ± 24	10.8%
**36–45**	86	0.0%	7.1 ± 1.9	153 ± 37	109 ± 25	34.9%
**> 45**	14	0.0%	6.3 ± 2.1	176 ± 39	118 ± 21	28.6%
	**n = 407**					

*PetCO*_*2*_ end-tidal carbon dioxide, *CO* cardiac output, *Sys. BP* systolic blood pressure.

## Discussion

### Emergently intubated trauma patients have critical injuries

All patients in the study required emergency endotracheal intubation following traumatic injury. The critical nature of these patients is expressed by the degree of severe anatomic injury, hemodynamic instability, major hemorrhage, abnormal pupils, metabolic acidosis, and death. Other investigators have also shown that trauma patients requiring emergency tracheal intubation are severely injured and have adverse outcomes [9,10].

### Reduced NICOM CO–clinical conditions

Low NICOM CO values were associated with eight clinically important conditions: ISS > 20, hypotension, bradycardia, major blood loss, RBC transfusion, abnormal pupils, cardiac arrest, and death. A lower CO with ISS > 20 was also found in our previous study which included non-ventilated patients, as well as those undergoing mechanical ventilation [1]. It is relevant that patients with an ISS > 20 in the current study had higher rates of hypotension, blood loss, and RBC transfusion, when compared to patients with ISS ≤ 20. These findings suggest that ISS > 20 patients had a greater degree of hemorrhage to explain their lower CO values. These findings are similar to observations demonstrated by the first author in a canine hemorrhagic shock model [11].

Patients with abnormal pupils had a lower CO and more hypotension than those with normal pupillary responses. Belzberg showed in a study of patients with severe head trauma that those with subsequent brain death initially had a decrease in cardiac index [12]. Because most patients with abnormal pupils were not in cardiac arrest, pupil dysfunction implies that other causes for brainstem dysfunction were present. Accordingly, our data results are in harmony with those of the Belzberg study. CO was also reduced in patients with bradycardia, a finding that seems to be intuitive. As well, it is instinctive that CO would be significantly lower or nil with cardiac arrest.

### Reduced PetCO_2_–clinical conditions

Both low PetCO_2_ and low CO were associated with the same eight clinical conditions: ISS > 20, hypotension, bradycardia, major blood loss, RBC transfusion, abnormal pupils, cardiac arrest, and death. In the current study, patients with ISS > 20 had a lower PetCO_2_ than those with an ISS ≤ 20. Because ISS > 20 patients had more hemodynamic instability and blood loss, it is likely the reduced PetCO_2_ with ISS > 20 is related to those factors. A study by Tyburski supports our findings when he showed that trauma patients undergoing emergency surgery and dying had more RBC transfusions, more hypotension, and lower end-tidal CO_2_ values [13].

A literature review produced four human investigations [2-5] and three animal studies [6-8] demonstrating a reduction in PetCO_2_ with hemodynamic instability. Of relevance, a large study of human cardiac arrest patients by Kolar demonstrated that PetCO_2_ was markedly decreased during cardiac arrest and substantially higher with return of spontaneous circulation [14]. The mean PetCO_2_ values during cardiac arrest of the current study and the Kolar investigation are virtually identical.

Patients with abnormal pupils, indicating brain stem dysfunction, had lower PetCO_2_ and greater hypotension. It seems likely that the lower PetCO_2_ may have been related to hemodynamic instability, which is common with traumatic brainstem dysfunction [15]. Furthermore, patients with abnormal pupils had a lower, critical GCS of four. It is also likely that the lower PetCO_2_ was due to therapeutic hyperventilation to manage suspected intracranial hypertension. However, minute ventilation volumes were not documented during the study.

### PetCO_2_ and NICOM CO relationship

This study demonstrated that low PetCO_2_ was associated low CO. Specifically, there was a highly significant direct relationship between PetCO_2_ and CO among the 318 paired values. Further, PetCO2 was significantly lower with CO < 4.5 L/min, when compared to CO ≥ 4.5 L/min. From a physiologic standpoint, a reduction in right ventricular CO leads to a decrease in pulmonary arterial blood flow, an increase in dead space, and a lower expired alveolar CO_2_ concentration [4,6,7].

There is substantial literature to suggest the PetCO_2_ and CO have a significant and direct relationship. A literature review produced only four human studies describing the relationship between PetCO_2_ and CO. In three of the studies, patients underwent cardiopulmonary bypass for cardiac surgery [2-4]. A study by Shibutani investigated patients with elective abdominal aortic aneurysm surgery [5]. All four studies described a significant decrease in PetCO_2_ as CO declined. The number of paired CO and PetCO_2_ values in the current study is larger, by far, in comparison to the other four human investigations. Of relevance, animal investigations of hemorrhagic shock [6,7] and cardiac arrest [8] describe sequential PetCO_2_ and CO monitoring prior to, during, and following hemodynamic instability. These three studies also demonstrated a significant direct relationship between PetCO_2_ and CO levels.

Of importance, a large study of human cardiac arrest patients (n = 737) demonstrated that PetCO_2_ was markedly decreased during cardiac arrest (6.9 ± 2.2) and substantially higher with return of spontaneous circulation (32.8 ± 9.1; *P* < 0.001) [14]. Although CO was not measured, CO would have been substantially higher with return of spontaneous circulation, in comparison to cardiac arrest. It seems reasonable that these observations also infer there is a positive relationship between PetCO_2_ and CO levels.

### Hypotensive patients

It is important to recognize that a substantial number of hypotensive trauma patients do not have major hemorrhage. However, control of significant bleeding is a quintessential principle in the management of critically injured patients. During hypotension, patients with major blood loss had a marked reduction in CO and PetCO_2_, when compared to hypotensive patients without major blood loss. This implies that CO and PetCO_2_ values may be helpful for clinical decision-making in managing hypotensive trauma patients.

Based on the findings of this study, a substantial reduction CO or PetCO_2_ in a hypotensive patient suggests there is major blood loss. Accordingly, the trauma surgeon would be impelled to administer red blood cells and secure hemorrhage control. That is, promptly proceed with celiotomy, thoracotomy, operative control of external hemorrhage, or arteriography and embolization, as clinically appropriate. An important caution is that reductions in CO or PetCO_2_ might also be related to non-hemorrhagic causes, e.g., cardiac contusion, cardiac tamponade, tension pneumothorax, or chronic heart failure. On the other hand, preservation of CO and PetCO_2_ during hypotension implies the absence of major blood loss. Therefore, the clinician would be more likely to obtain an urgent CT scan to identify or exclude occult torso bleeding.

### Potential value of cardiac function monitoring

Literature documentation is evolving to suggest that CO, stroke volume index, and dynamic cardiac preload monitoring may be useful to optimize cardiac function during stress and hemodynamic instability. Marik presented the evidence suggesting that dynamic cardiac preload variables during mechanical ventilation are highly accurate in predicting volume responsiveness [16]. Other investigators have demonstrated the value of using stroke volume variation to determine intraoperative fluid administration during high risk surgery [17]. Marik has also suggested a goal-directed resuscitation protocol, using stroke volume, stroke volume index, cardiac index, and their response to fluid infusion [18].

The noninvasive, user-friendly features of NICOM are appealing for managing time-pressured, critical trauma patients in the Emergency Department. In our previous publication, we demonstrated that 90% of patients had an initial CO within 8.5 minutes of Emergency Department arrival [1]. The compelling literature and the user-friendly aspects of NICOM suggest that monitoring cardiac function during Emergency Department trauma activation may become more widespread.

### NICOM CO validity

This study indicates that NICOM CO values are valid and nonrandom in trauma patients undergoing emergency tracheal intubation. Clinical discrimination is demonstrated by the significant reduction in CO with increased ISS, abnormal pupils, major blood loss, RBC transfusion, death, and hypotensive patients with major blood loss. Physiologic validity is suggested by the statistically lower CO with hypotension, bradycardia, cardiac arrest, and reduced PetCO_2_ levels. In our previous study, we found multiple statistical relationships of CO with 15 patient clinical conditions. These findings imply that NICOM provides an objective and clinically valid, reliable (nonrandom), relevant, and discriminate measure of cardiac function in acutely injured trauma activation patients [1].

NICOM CO values have been shown to have acceptable correlations with paired values obtained using other methodologies, as summarized by Marik [18]. Although the pulmonary artery catheter thermodilution technique may be considered a “practical” gold standard, the procedure has limitations [19]. Additionally, the pulmonary artery catheter thermodilution technique has an inherent error rate of 10% to 20%, when compared to less practical, gold standard methods (Fick and dye-dilution) [19]. We think that clinical-validation studies may complement, and possibly be more important than, other studies that focus on accuracy comparison between different CO methodologies.

Limitations of the study must be considered. A larger study might demonstrate different mean NICOM CO and PetCO_2_ values than found in the current investigation. We did not compare NICOM CO’s to a gold standard cardiac output methodology. We believe the most reliable indicator of cardiac output is the Fick and dye-dilution, however, this is impractical to perform in the acute setting. Although debatable, we believe it was reasonable to assume that the CO was zero in patients who presented with asystole. However, the impact of chest compressions on CO is uncertain. Because PetCO_2_ was not always zero, it is likely that some CO was generated with chest compressions. Table 4 demonstrated a trichotomous distribution of mortality for low, intermediate, and high PetCO_2_ ranges (Table 4). Specifically, we have no clear explanation for the increase in mortality with PetCO_2_ ≥ 36. Perhaps, a larger cohort with detailed analyses might provide insight as to why there was a trichotomous distribution of mortality, according to the different PetCO_2_ ranges. Because PetCO_2_ can be affected by minute ventilation volume, our failure to document and assess this parameter is a study limitation.

## Conclusions

The need for emergency tracheal intubation following trauma signifies the presence of critical injuries and adverse outcomes. In critically injured trauma patients, hypotension without major blood loss is not uncommon. PetCO_2_ and NICOM CO values may help to discriminate patients without and with major blood loss. A statistically significant relationship exists between low PetCO_2_ and low CO. This relationship is buttressed by the observation that NICOM CO and PetCO_2_ values each decreased under the same clinical conditions: hemodynamic instability, hemorrhage, abnormal pupils, and death. Similar to findings in the cardiac arrest literature, PetCO_2_ levels were markedly reduced with cardiac arrest. The study indicates that NICOM CO values, in critically injured trauma patients, are clinically discriminate and have physiologic validity. Based on the noninvasive and user-friendly nature of NICOM, the evolving literature, and this study, CO monitoring may prove to be useful in optimizing cardiac function and organ perfusion in critically injured trauma patients. This study also suggests that there may be value in the continuous Emergency Department PetCO_2_ monitoring of trauma patients requiring emergency tracheal intubation.

### Key messages

•The study includes critically injured trauma patients requiring emergency tracheal intubation and includes, by far, the largest number of paired cardiac output and end-tidal CO_2_ data points in humans, when compared to the published literature.

•Decreases in NICOM cardiac output values were statistically associated with reductions in end-tidal CO_2_.

•Both low CO and low end-tidal CO_2_ values were associated with the same eight patient-risk conditions, thus providing clinical support for the statistical relationship between cardiac output and end-tidal CO_2_.

•Hypotensive patients with major blood loss had clinically discernible and statistically significant reductions in cardiac output and end-tidal CO_2_, when compared to hypotensive patients without major blood loss.

•The study indicates that noninvasive cardiac output (NICOM) values, in critically injured trauma patients are clinically discriminate and have physiologic validity.

## Abbreviations

BP: Blood pressure; CO: Cardiac output; ISS: Injury severity score; NICOM: Noninvasive cardiac output monitor; PetCO2: End-tidal carbon dioxide; RBC: Red blood cell.

## Competing interests

The authors declare that they have no competing interests.

## Authors’ contributions

CMD conceptualized and designed the study. CMD, TJC, BSG, JPF, JAM, BL, and BH were involved in the day-to-day oversight of the study. CMD, TJC, JPF, JAM, BH, and RM performed the data collection. CMD, TJC, and BSG performed the data analysis. CMD, TJC, BSG, JPF, JAM, BL, BH, and RM performed the data interpretation. CMD and TJC performed the literature search and drafted the manuscript. CMD, TJC, BSG, JPF, JAM, BL, BH, and RM critically revised the manuscript for important intellectual content. All authors made substantial contributions to conception and design, or acquisition of data, or analysis and interpretation of data. All authors have been involved in drafting the manuscript or revising it critically for important intellectual content. All authors read and approved the final manuscript.

## Meeting presentation

Annual meeting of the American Association for the Surgery of Trauma, in Kauai, Hawaii, September 12-15, 2012.

## Pre-publication history

The pre-publication history for this paper can be accessed here:

http://www.biomedcentral.com/1471-2253/13/20/prepub
